# A crowdsourcing approach to track the expansion of the brown marmorated stinkbug *Halyomorpha halys* (Stål, 1855) in France

**DOI:** 10.3897/BDJ.9.e66335

**Published:** 2021-05-19

**Authors:** Marguerite Chartois, Jean-Claude Streito, Éric Pierre, Jean-Marc Armand, Jonathan Gaudin, Jean-Pierre Rossi

**Affiliations:** 1 UMR CBGP, INRAE, CIRAD, IRD, Montpellier SupAgro, Montpellier, France UMR CBGP, INRAE, CIRAD, IRD, Montpellier SupAgro Montpellier France; 2 UMR SAVE INRAE Bordeaux Science Agro, ISVV, Bordeaux, France UMR SAVE INRAE Bordeaux Science Agro, ISVV Bordeaux France

## Abstract

**Background:**

*Halyomorpha halys* (Stål, 1855), the brown marmorated stinkbug (BMSB) is a highly successful invasive species, native to eastern Asia. It has managed to spread into North America and Europe in recent decades, causing severe damage to various crops. BMSB has been detected in Europe in 2004 and has since expanded in more than 20 countries from Sweden to Greece and Spain to Turkey, the South European Territory of Russia (Krasnodar region) and Abkhazia. In 2012, we set up a citizen science survey to monitor BMSB expansion in France.

**New information:**

The present crowdsourcing survey was initiated in 2012 and provided a large number of occurrence points of BMSB. These data allowed to track the expansion of the species in France from 2012 to 2019 and brought information about its phenology and distribution in various habitats. The dataset comprises both valid and invalid sightings, thereby allowing us to examine changes in the quality of citizen reports during the course of the survey. Despite a large proportion of misidentifications, the survey provided a large number of valid occurrences. Furthermore, valuable information on hemipterans of Pentatomidae and Coreidae families entering habitations were also gathered. The dataset also illustrates that, although designed for a large public, the Agiir application was mostly used by urban dwellers with very few sightings stemming from professionals of agricultural sectors.

## Introduction

In Europe, two species of the family Pentatomidae are listed as invasive, namely *Halyomorpha halys* and *Nezara viridula* (McPherson 2018). *Halyomorpha halys* (Stål, 1855), the brown marmorated stinkbug (BMSB) is native to eastern Asia and is common in the temperate regions of China, Japan and the Republic of Korea (Hamilton et al. 2018). It has been reported in North America in 2001 and in Europe in 2004 where it quickly expanded in more than 20 countries, as well as in the South European Territory of Russia and in Abkhazia (Bergmann et al. 2016, Gapon 2016).

BMSB was first reported from France in 2012 in the suburb of Strasbourg (Eastern France) (Callot and Brua 2013) and has since colonised large parts of the country. We developed a citizen science operation at the beginning of the invasion (Streito et al. 2014) and we report here the data (Suppl. material 1) that were gathered between 2012 and 2019. Our crowdsourcing survey is based on a smartphone application named Agiir allowing volunteers to report cases of BMSB observations. The data are stored in a database and further carefully checked by professional entomologists. We here provide all reports, either valid BMSB observations or erroneous sightings in order to provide the entire dataset generated in the course of the survey.

## Sampling methods

### Sampling description

The survey was based on a smartphone application named Agiir (Alerter Gérer les Insectes Invasifs et/ou Ravageurs) developed at our institute Inrae, aiming at providing information about harmful invasive insect species and collecting citizen reports in France. The system is backed by a website providing species diagnostic tools including pictures and morphological description, allowing BMSB identification along with comprehensive information about its biology (https://ephytia.inrae.fr/fr/P/128/Agiir). Volunteers reported their sightings by means of their smartphone (date, coordinates as decimal degrees and the name of the observer are automatically filled in by the phone application). The application is available for smartphones or touch tablets and is distributed free of charge. It was advertised through various channels including national publication in French (Streito et al. 2014), Inrae institutional communication (website) or entomologist networks, such as "Le monde des insectes" (www.insecte.org). Our survey thus consisted of “passive monitoring” that is an approach where scientists did not actively collect data, but rather relied on citizen contribution. The data were stored in a database, examined and confirmed by entomologists. The survey started on 15 August 2012.

Two additional sources of data were gathered alongside the Agiir system: a) extra-Agiir citizen reports received by phone or email and b) BMSB reports from "naturalist" networks. Naturalists refer to a special category of contributors having amateur or advanced knowledge in the field of entomology. Their observations were directly reported to us by phone or emails or through different entomologist networks, for example, Le monde des insectes (www.insecte.org). JCS collected these reports from 15/08/2012 until the end of 2019 and checked specimen identification, dates and spatial location. It should be noted that Agiir reports, generated by naturalists, could not be distinguished from other reports.

Without duplicated records, the dataset contained a total of 4002 reports, 3538 from Agiir application, 377 citizen direct reports and 87 extra-Agiir naturalist reports.

**Species identification**

**Figure 1.**
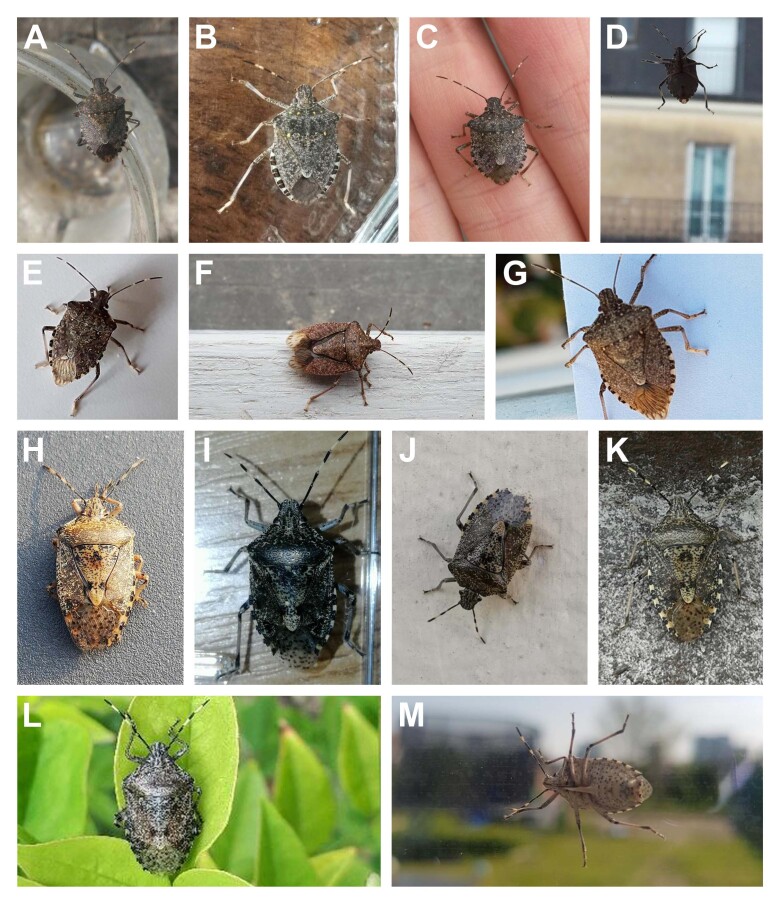
A set of pictures illustrating the criteria allowing the identification of *Halyomorpha halys* and *Rhaphigaster nebulosa*, two species frequently confused by citizen science contributors. **A-G.** valid reports of *H. halys* checked using the following criteria: antennae segment V and IV with white rings facing each other, elongated dark spots on the fore wing membrane, no spine on the underside base of abdomen and light callus along scutellum base; **H-M.** invalid reports where *H. halys* is confused with *Rhaphigaster nebulosa*. *R. nebulosa* is identified on the basis of the following criteria: antennae segment V and IV with white rings separated by a dark ring, rounded dark marks on the fore wing membrane, no light callus on scutellum base, spine on the underside base of abdomen.

**Figure 2.**
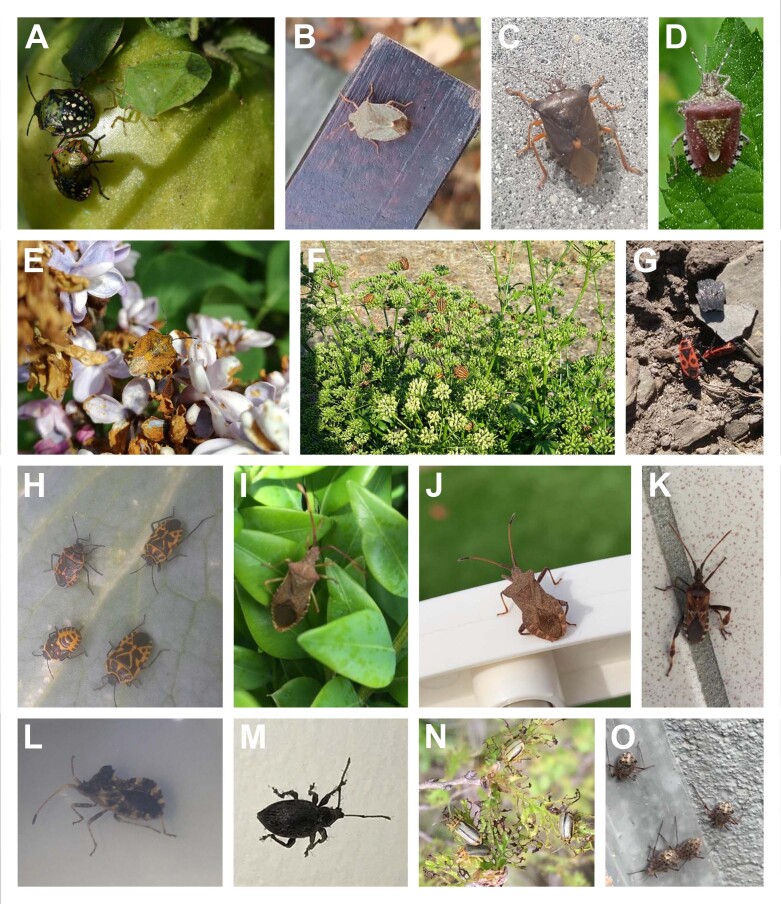
Arthropod diversity reported by citizen science volunteers. **A-L.** Various Pentatomids frequently entering habitations or observed in gardens: **A.** *Nezara viridula* adult and nymph; **B.** *Palomena prasina*; **C.** *Pentatoma rufipes*; **D.** *Dolycoris baccarum*; **E.** *Carpocoris* sp.; **F.** *Graphosoma italicum*; **G.** *Pyrrhocoris apterus*; **H.** *Eurydema* sp. adult and nymph; **I-L.** Various species of Coreidae family; **I.** *Gonocerus acuteangulatus*; **J.** *Coreus marginatus*; **K.** *Leptoglossus occidentalis*; **L.** *Centrocoris* sp.; **M-O.** Other insects: **M.** Coleoptera Curculionidae; **N.** Coleoptera Chrysomelidae (*Xanthogaleruca luteola*); **O.** Hemiptera Aphididae.

**Figure 3.**
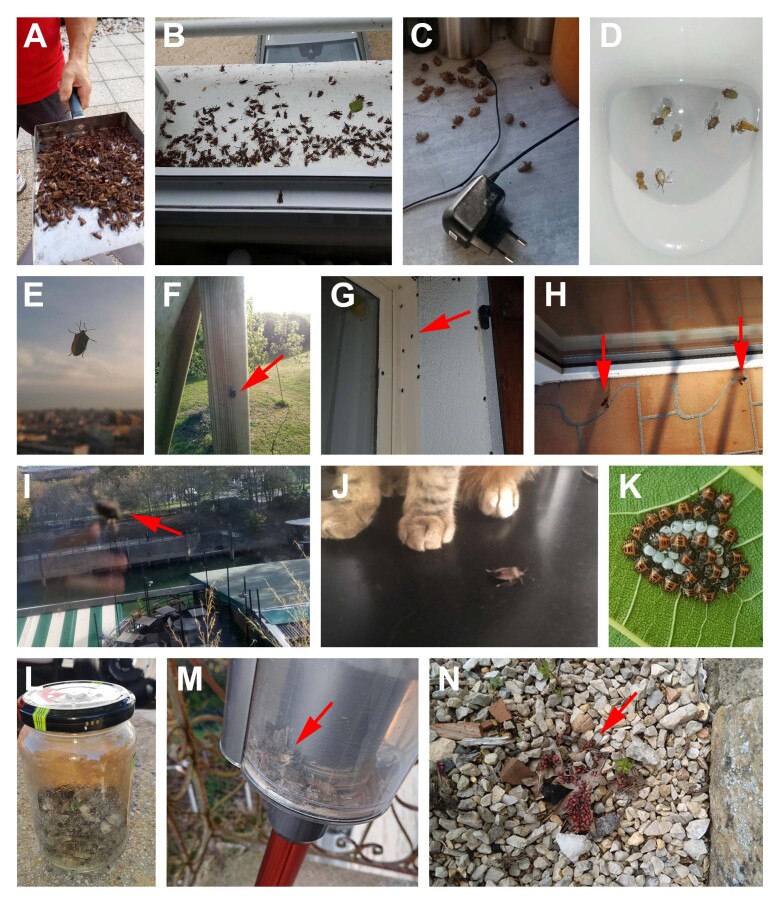
Examples of reports associated to poor picture definition. **A-B.** Where species identification was possible despite poor picture definition (*Leptoglossus occidentalis*); **C-D.** Difficult species identification (reports labelled Pentatomidae in the dataset); **E-H.** Specimen too far away and picture definition too low: taxonomic details barely visible (reports labelled Pentatomidae in the dataset); **I-J.** Blurry pictures: taxonomic details barely visible (reports labelled Pentatomidae in the dataset); **K.** Early development stages of Pentatomidae larvae, difficult specific identification; **L-N** Indistinguishable specimens.

Species identification relied on the picture associated with the records (Figs 1, 2, 3). Valid records were associated with the label "*Halyomorpha halys*" in the field named "scientific_name". Invalid records corresponded to different situations.

1. Record associated with no picture or poor quality picture that did not allow reliable identification (picture definition too low, blurry picture, truncated picture or unzoomed picture. These cases were associated with labels "no picture" or "poor picture definition" in the "issues" field. No picture implied no identification. In the case of low-quality pictures, we identified the specimen at the best taxonomic possible level.
2. Reports on eggs, exuvia or young nymphs for which formal identification is difficult were listed as Pentatomidae in the field "scientific_name".
3. Some reports were associated with screenshots instead of phone pictures. The problem is that such reports might not be illustrated with the picture of the reported specimen, but a picture considered to be representative of the specimen by the contributor. For example, one report showed a screenshot of the Wikipedia page associated of *Leptoglossus occidentalis* (https://fr.wikipedia.org/wiki/Leptoglossus_occidentalis). Such highly unreliable reports were associated with the modality "screenshot" in the "issues" field.

Some sightings revealed other species, generally other pentatomids, such as the mottled shieldbug *Rhaphigaster nebulosa* which is commonly mistaken for the BMSB in France (Streito et al. 2014). Other species included *Nezara viridula*, *Dolycoris baccarum*, *Palomena prasina* or *Pentatoma rufipes*. The western conifer seed bug *Leptoglossus occidentalis* (Coreidae) was frequently reported as well. In certain cases, volunteers reported several hemipteran species at once, in addition to BMSB or not. These species were reported in the "additionnal_species" field. Finally, duplicated records i.e. those sent on several occasions to the application with identical picture were labelled "duplicated form" in the "issues" field. For each duplicate, the id of the corresponding duplicated record is reported in the "initial_duplicated_form" field.

As for Agiir reports without picture, but with potentially important information (e.g. occurrence in a previously unoccupied region), the taxonomic identification was checked by contacting the contributor in order to get either a picture or a specimen. At the beginning of the BMSB expansion in France i.e. 2012-2013, all Agiir records came from naturalists and were checked by specimen examination. We later validated reports on the basis of pictures.

**Figure 4.**
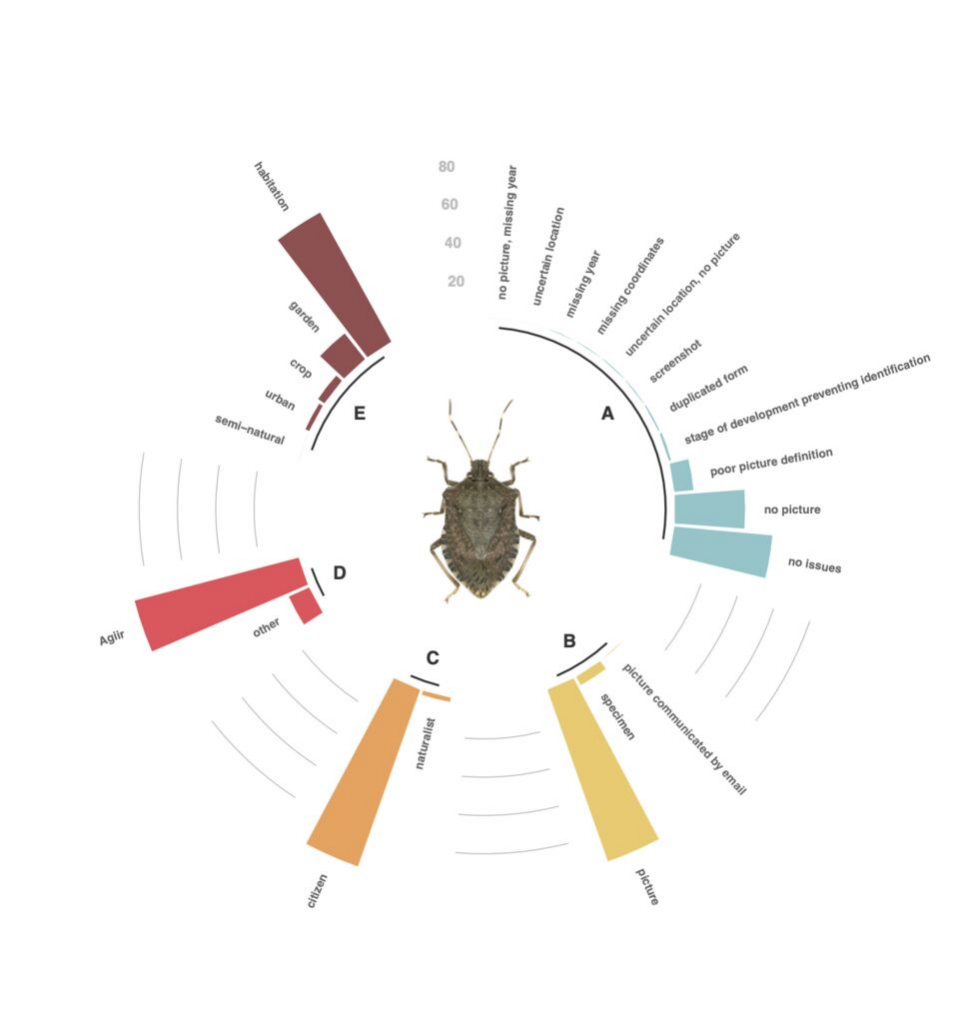
Composition of the dataset. **A.** Issues associated to volunteer reports; **B.** Nature of support for specimen identification; **C.** Proportion of citizen and naturalist contributions; **D.** Proportion of medias contribution: French network Agiir and direct reports; **E.** Distribution of sightings in the various environments considered in the dataset.

Fig. 4 displays the list and frequency of issues encountered in the dataset.

**Environment and phenology**

**Figure 5.**
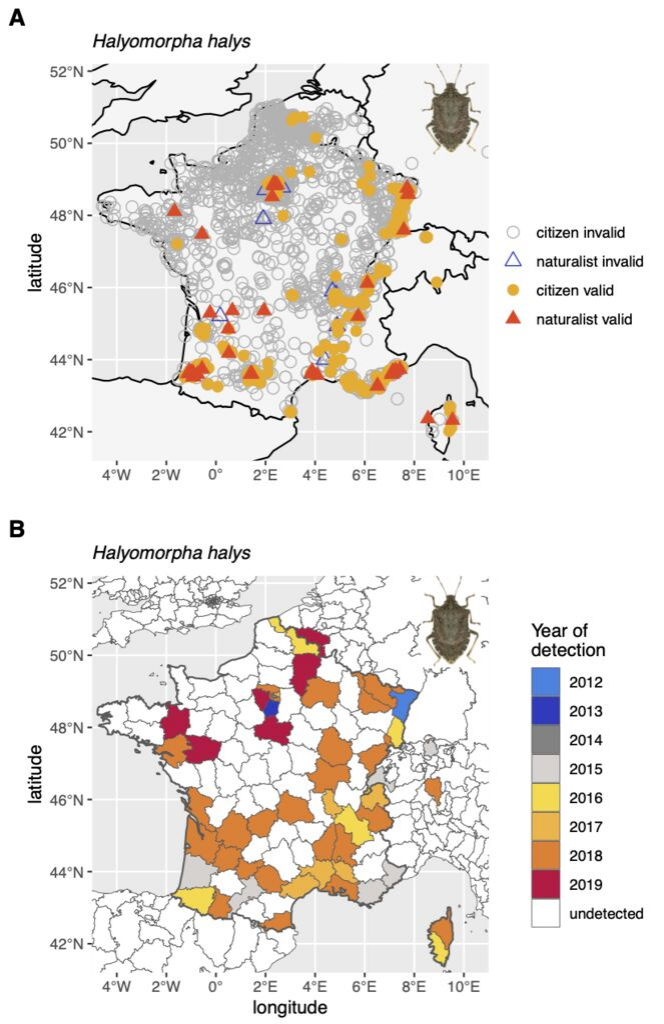
Occurrence data of *Halyomorpha halys* collected in France between 2012 and 2019. **A.** Distribution of citizen and naturalist contributions; **B.** Geographical expansion of the BMSB from 2012 to 2019.

A standardised description of the environment where the specimen was observed is available within the Agiir system. It comprises three modalities: "habitation", "garden" and "crop" and appears in the "environment" field. Direct reports (extra-Agiir) were associated with a more detailed environment description that are reported in the "verbatim_environment" field. The latter description was simplified and reported in the "environment" field. As shown in Figure 5, most of the reports occur inside habitations and contributors were mostly urban dwellers reporting domestic nuisances of BMSB entering houses in October. Very few sightings were gathered from the professionals of agricultural sectors.

**Additional information**

Each Agiir record was associated with the location of the observation through the longitude and latitude coordinates provided by the Global Positioning System (GPS) device of the smartphone system. Standards coordinates provided in decimal degrees correspond to the World Geodetic System WGS 84 aka European Petroleum Survey Group EPSG 4326 (https://spatialreference.org/ref/epsg/wgs-84/). Some extra-Agiir reports missed spatial coordinates, but were associated with precise locality names. Coordinates were manually deduced by positioning the occurrence at the centroid of the locality.

## Geographic coverage

### Description

The survey was initially intended to generate observational data in France, but citizens of other countries could contribute, in particular volunteers from neighbouring francophone countries (e.g. some sightings were associated with longitude/latitude coordinates pointing to Canada). Certain reports corresponded to locations falling in the sea and/or in countries far away from the known BMSB range (e.g. Niger, Benin, Cameroun) and were thus labelled "uncertain location" in the "issues" field. Distribution of occurrences gathered by citizens and naturalists is displayed in Fig. 5, along with geographic expansion of BMSB.

## Taxonomic coverage

### Description

**Figure 6.**
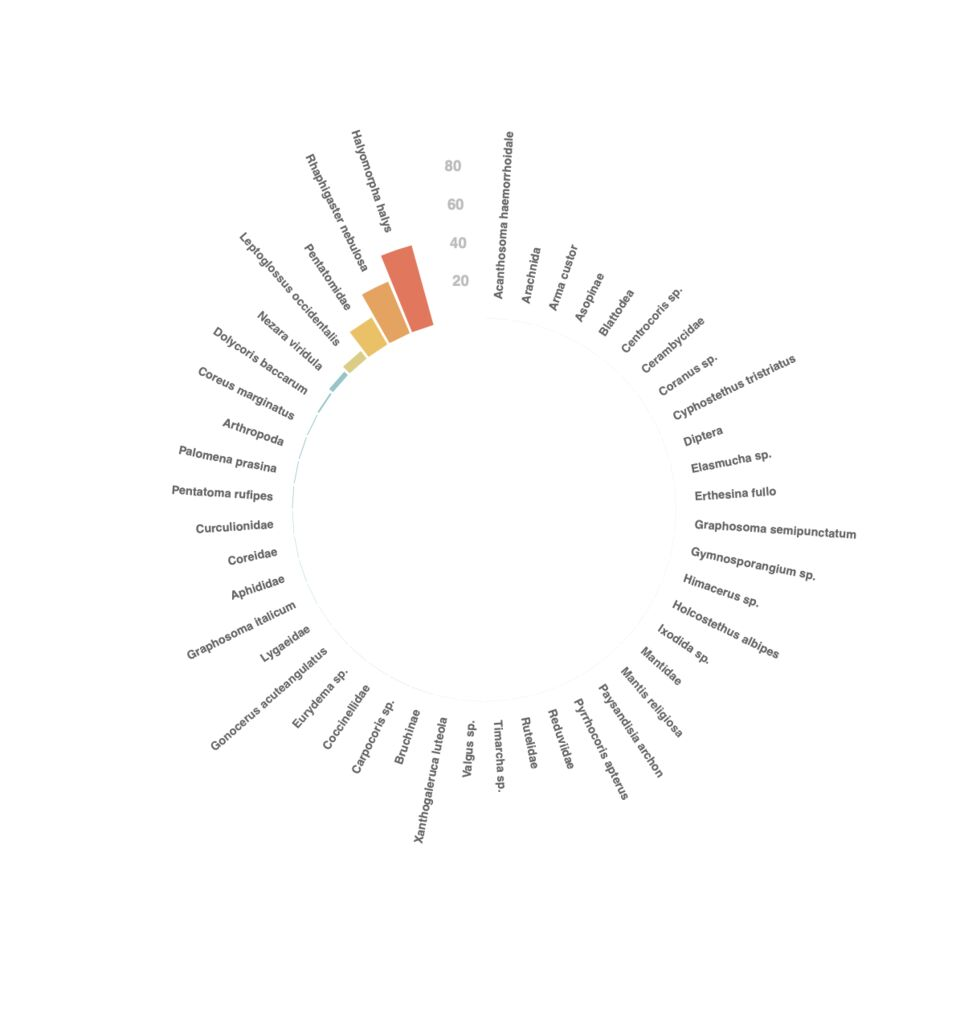
Barplot showing the taxonomic diversity of the arthropods reported by citizen volunteers.

The study concerns the brown marmorated stink bug *Halyomorpha halys* (Stål, 1855) (Hemiptera: Pentatomidae). Data about other species were also collected: bugs of the Pentatomidae or Coreidae family, various arthropods (e.g. Coccinellidae, Aphididae, Cerambycidae, Curculionidae, Diptera) and arachnids. The diversity of reported organisms is listed in Fig. 6 and depicted in Figs 1, 2, 3. An occurrence of *Erthesina fullo*, an invasive species not currently established in France, was recorded. It corresponded to a single specimen observed in a postal parcel. This sighting is assimilated to an interception with no proof of establishment.

## Temporal coverage

### Notes

Data collection started on 15 August 2012 and is on-going. We here report data collected between 15 August 2012 and 31 December 2019.

## Usage licence

### Usage licence

Creative Commons Public Domain Waiver (CC-Zero)

## Data resources

### Data package title

A crowdsourcing approach to track the expansion of the brown marmorated stinkbug *Halyomorpha halys* (Stål, 1855) in France.

### Number of data sets

1

### Data set 1.

#### Data set name

A crowdsourcing approach to track the expansion of the brown marmorated stinkbug *Halyomorpha halys* (Stål, 1855) in France.

#### Data format

csv

#### Number of columns

14

#### Data format version

csv

#### Description

The dataset provides a large number of occurrences of *Halyomorpha halys* from 2012 to 2019 (Suppl. material 1). Data were gathered in the framework of a crowdsourcing survey aiming at tracking the expansion of *H. halys* in France. The dataset comprises both valid and invalid reports. The dataset describes (i) the diversity of arthropods sightings, (ii) the date and spatial location of the observation, (iii) some details about the environment where each observation was made, (iv) the support from which the reported specimens could be identified and (v) the issues associated with each report.

**Data set 1.** 

Column label	Column description
id	unique identifier of the report; each row of the file corresponds to a report.
latitude	geographic coordinates in decimal degrees (World Geodetic System WGS 84 aka European Petroleum Survey Group EPSG 4326). Number rounded to 3 decimal places.
longitude	geographic coordinates in decimal degrees (World Geodetic System WGS 84 aka European Petroleum Survey Group EPSG 4326). Number rounded to 3 decimal places.
scientific_name	scientific name of the reported specimen. NA denotes reports for which identification was impossible. Modalities: NA; *Acanthosoma haemorrhoidale*; Aphididae; Arachnida; *Arma custor*; Arthropoda; Asopinae; Blattodea; Bruchinae; *Carpocoris* sp.; *Centrocoris* sp.; Cerambycidae; Coccinellidae; *Coranus* sp.; Coreidae; *Coreus marginatus*; Curculionidae; *Cyphostethus tristriatus*; Diptera; *Dolycoris baccarum*; *Elasmucha* sp.; *Erthesina fullo*; *Eurydema* sp.; *Gonocerus acuteangulatus*; *Graphosoma italicum*; *Gymnosporangium* sp.; *Halyomorpha halys*; *Himacerus* sp.; *Holcostethus albipes*; *Ixodida* sp.; *Leptoglossus occidentalis*; Lygaeidae; Mantidae; *Mantis religiosa*; *Nezara viridula*; *Palomena prasina*; *Paysandisia archon*; *Pentatoma rufipes*; Pentatomidae; *Pyrrhocoris apterus*; Reduviidae; *Rhaphigaster nebulosa*; Rutelidae; *Timarcha* sp.; *Valgus* sp.; *Xanthogaleruca luteola*.
additional_species	scientific name of additional specimens present in the picture. NA denotes reports with no additional species. Modalities: NA; *Coreus marginatus*; *Graphosoma semipunctatum*; *Leptoglossus occidentalis*; *Nezara viridula*; *Palomena prasina*; *Pentatomidae*; *Rhaphigaster nebulosa*.
date	date of observation (day/month/year). NA denotes reports with missing year.
verbatim_environment	description of the environment where the extra-Agiir observations were made. NA denotes reports with no description of the environment. Modalities: NA; agricultural area; apple tree; boat; culture; garden; habitation; kiwi orchard; lemon tree; peri-urban area; Pittosporum sp.; private garden; rose tree; sidewalk; virginia creeper.
environment	environment description available within the Agiir application plus simplified description of the environment of the extra-Agiir reports (verbatim_environment above). NA denotes reports with no description of the environment. Modalities: NA; crop; garden; habitation; urban.
identification_support	support for specimen identification. NA denotes reports with missing picture. Modalities: NA; picture; picture communicated by email; specimen.
issues	warnings about specimen observation or taxonomic identification. Modalities: duplicated form; missing coordinates; missing year; no issues; no picture; no picture, missing year; poor picture definition; screenshot; stage of development preventing identification; uncertain location; uncertain location, no picture.
initial_duplicated_form	id of the corresponding duplicated record. NA denotes non-duplicated reports.
source	status of citizen contributor. Modalities: citizen; naturalist.
media	origin of the report. Modalities: Agiir; other.
note	additional commentary

## Supplementary Material

F515AAE2-4001-56AC-8C64-14270B8B1F4C 10.3897/BDJ.9.e66335.suppl1 Supplementary material 1 A crowdsourcing approach to track the expansion of the brown marmorated stinkbug *Halyomorpha halys* (Stål, 1855) in France Data type Occurrences Brief description The dataset provides a large number of occurrences of *Halyomorpha halys* from 2012 to 2019. Data were gathered in the framework of a crowdsourcing survey aiming at tracking the expansion of *H. halys* in France. The dataset comprises both valid and invalid reports. The dataset describes (i) the diversity of arthropods sightings, (ii) the date and spatial location of the observation, (iii) some details about the environment where each observation was made, (iv) the support from which the reported specimens could be identified and (v) the issues associated with each report. File: oo_522847.csv https://binary.pensoft.net/file/522847 Marguerite Chartois, Jean-Claude Streito, Éric Pierre, Jean-Marc Armand, Jonathan Gaudin & Jean-Pierre Rossi
